# Supplementary material for: Strand-Specific RNA Sequencing Reveals Gene Expression Patterns in F1 Chick Breast Muscle and Liver after Hatching
Source: Animals (Basel). 2024 Apr 29;14(9):1335. doi: 10.3390/ani14091335 (PMC11083249; doi:10.3390/ani14091335)
Supplement: Supplementary file 1 [file animals-14-01335-s001.zip › animals-2958164-supplementary.pdf]

animals

MDPI

Table S1. The quality control data of the newly sequenced transcriptome data of parental lines.

Sample	Raw Reads	Bases(GB)	GC(%)	Q20(%)	Q30(%)	Avg.Quality
Maternal line-liver-female-1	38256548	5.738	49.435	94.755	88.86	34.93
Maternal line-liver-female-2	37231242	5.585	48.925	94.9	89.145	34.985
Maternal line-liver-female-3	38164492	5.725	49.69	95.685	90.08	35.205
Maternal line-liver-male-1	39384692	5.908	48.4	94.825	89.125	34.97
Maternal line-liver-male-2	37623608	5.644	48.99	95.44	89.87	35.145
Maternal line-liver-male-3	39594696	5.939	48.52	95.81	90.375	35.26
Maternal line-muscle-female-1	39072190	5.861	51.83	95.03	89.455	35.04
Maternal line-muscle-female-2	37673436	5.651	51.665	94.655	88.67	34.895
Maternal line-muscle-female-3	41229444	6.184	51.28	95.69	90.36	35.24
Maternal line-muscle-male-1	42568778	6.385	51.44	95.435	90.035	35.165
Maternal line-muscle-male-2	36514700	5.477	51.585	94.4	88.67	34.855
Maternal line-muscle-male-3	37119460	5.568	52.24	95.27	89.75	35.105
Paternal line-liver-female-1	40724868	6.109	48.695	95.4	89.965	35.155
Paternal line-liver-female-2	89272664	13.391	48.06	95.965	90.425	35.285
Paternal line-liver-female-3	40022266	6.003	49.225	95.925	90.585	35.3
Paternal line-liver-male-1	56154980	8.423	48.515	96.54	91.62	35.51
Paternal line-liver-male-2	39479244	5.922	53.66	93.555	87.695	34.625
Paternal line-liver-male-3	37987870	5.698	49.855	95.205	89.385	35.055
Paternal line-muscle-female-1	39319988	5.898	52.605	94.685	88.59	34.885
Paternal line-muscle-female-2	37766468	5.665	50.84	94.97	89.005	34.98
Paternal line-muscle-female-3	36359676	5.454	51.45	94.79	89.045	34.955
Paternal line-muscle-male-1	37438142	5.616	51.93	95.445	89.81	35.135
Paternal line-muscle-male-2	42584706	6.388	50.435	95.83	90.68	35.295
Paternal line-muscle-male-3	38257538	5.739	49.98	96.01	90.76	35.335

Table S2. Significantly enriched GO terms of different gene sets in the breast muscle.

**Gene set**	**Category**	**Term**	**Gene count**	***p*-value**	**Fold Enrichment**
Maternal line vs. F1 cross	BP	GO:0030866~cortical actin cytoskeleton organization	7	4.95E-04	6.372293447
	BP	GO:0006805~xenobiotic metabolic process	9	0.004597979	3.351660839
	BP	GO:0042073~intraciliary transport	6	0.004713452	5.174493927
	BP	GO:0006397~mRNA processing	11	0.00565768	2.772998028
	BP	GO:0006869~lipid transport	7	0.017905476	3.277179487
	BP	GO:0032784~regulation of DNA-templated transcription, elongation	3	0.020498708	12.28942308
	BP	GO:0006412~translation	14	0.021257127	2.012303194
	BP	GO:0070328~triglyceride homeostasis	4	0.025746286	5.958508159
	BP	GO:0008053~mitochondrial fusion	4	0.025746286	5.958508159
	BP	GO:0000122~negative regulation of transcription from RNA polymerase II promoter	27	0.02792995	1.541530421
	BP	GO:0042060~wound healing	6	0.029131647	3.390185676
	BP	GO:0045778~positive regulation of ossification	3	0.032794175	9.831538462
	BP	GO:0006939~smooth muscle contraction	4	0.032805327	5.461965812

	BP	GO:0042632~cholesterol homeostasis	7	0.040485459	2.730982906
	BP	GO:0030490~maturation of SSU-rRNA	4	0.040761384	5.041814596
	BP	GO:0000413~protein peptidyl-prolyl isomerization	5	0.041641434	3.724067599
	BP	GO:0032981~mitochondrial respiratory chain complex I assembly	6	0.042569936	3.072355769
	BP	GO:0006357~regulation of transcription from RNA polymerase II promoter	72	0.046735774	1.231507949
	BP	GO:0008585~female gonad development	4	0.049592535	4.681684982
	BP	GO:0042744~hydrogen peroxide catabolic process	4	0.049592535	4.681684982
	BP	GO:0042554~superoxide anion generation	4	0.049592535	4.681684982
	CC	GO:0005840~ribosome	16	0.001697472	2.50235728
	CC	GO:0043231~intracellular membrane-bounded organelle	17	0.006627658	2.108012583
	CC	GO:0030992~intraciliary transport particle B	5	0.008908804	5.786701209
	CC	GO:0005747~mitochondrial respiratory chain complex I	7	0.025617494	3.038018135
	CC	GO:0005930~axoneme	7	0.031802196	2.893350604
	CC	GO:0005929~cilium	10	0.039739739	2.170012953
	CC	GO:1902494~catalytic complex	3	0.042469282	8.680051813
	CC	GO:0022625~cytosolic large ribosomal subunit	7	0.042772815	2.700460564
	MF	GO:0001228~transcriptional activator activity, RNA polymerase II transcription regulatory region sequence-specific binding	20	0.013269432	1.827500122
	MF	GO:0003755~peptidyl-prolyl cis-trans isomerase activity	7	0.014822576	3.417425228
	MF	GO:0001046~core promoter sequence-specific DNA binding	5	0.014956458	5.025625335
	MF	GO:0003735~structural constituent of ribosome	16	0.017806853	1.938964668
	MF	GO:0019825~oxygen binding	5	0.018359454	4.746423927
	MF	GO:0001222~transcription corepressor binding	5	0.018359454	4.746423927
	MF	GO:0008146~sulfotransferase activity	6	0.018580909	3.797139142
	MF	GO:0004879~RNA polymerase II transcription factor activity, ligand-activated sequence-specific DNA binding	8	0.022853796	2.78973488
	MF	GO:0005102~receptor binding	14	0.036414969	1.868904421
	MF	GO:0003682~chromatin binding	19	0.036726004	1.66489947
	MF	GO:0004896~cytokine receptor activity	7	0.037633524	2.781625185
	MF	GO:0016887~ATPase activity	25	0.038780938	1.525636262
	MF	GO:0004497~monooxygenase activity	5	0.042216921	3.714592639
	MF	GO:0020037~heme binding	12	0.045251492	1.934391638
Paternal line vs. F1 cross	BP	GO:0030866~cortical actin cytoskeleton organization	6	0.002545125	5.933611885
	BP	GO:0008284~positive regulation of cell proliferation	18	0.004132833	2.150436522
	BP	GO:0006082~organic acid metabolic process	6	0.004195423	5.340250696
	BP	GO:0002181~cytoplasmic translation	7	0.007904906	3.893932799
	BP	GO:0006805~xenobiotic metabolic process	8	0.010599168	3.236515574
	BP	GO:0008625~extrinsic apoptotic signaling pathway via death domain receptors	4	0.011447017	7.911482513

	BP	GO:0008219~cell death	4	0.020674525	6.473031147
	BP	GO:0006412~translation	13	0.026013916	2.029919855
	BP	GO:0055085~transmembrane transport	21	0.031677126	1.639550652
	BP	GO:0031295~T cell costimulation	4	0.032967653	5.477180201
	BP	GO:0001678~cellular glucose homeostasis	4	0.032967653	5.477180201
	BP	GO:0006606~protein import into nucleus	8	0.036116849	2.542976522
	BP	GO:0042130~negative regulation of T cell proliferation	4	0.040252869	5.085953044
	BP	GO:0042744~hydrogen peroxide catabolic process	4	0.040252869	5.085953044
	BP	GO:0032874~positive regulation of stress-activated MAPK cascade	3	0.040539257	8.900417827
	BP	GO:0006952~defense response	6	0.044085126	3.051571827
	BP	GO:0033617~mitochondrial respiratory chain complex IV assembly	4	0.048276955	4.746889508
	CC	GO:0030141~secretory granule	7	0.005181377	4.244401213
	CC	GO:0005840~ribosome	14	0.00593127	2.370746623
	CC	GO:0005615~extracellular space	63	0.006251384	1.394803225
	CC	GO:0043231~intracellular membrane-bounded organelle	15	0.016808438	2.013925065
	CC	GO:0072562~blood microparticle	5	0.019373059	4.699158485
	MF	GO:0005506~iron ion binding	17	2.02E-04	2.922291994
	MF	GO:0020037~heme binding	16	7.09E-04	2.724445366
	MF	GO:0005504~fatty acid binding	6	0.004956746	5.156985871
	MF	GO:0005319~lipid transporter activity	5	0.007771085	6.016483516
	MF	GO:0016712~oxidoreductase activity, acting on paired donors, with incorporation or reduction of molecular oxygen, reduced flavin or flavoprotein as one donor, and incorporation of one atom of oxygen	6	0.010779907	4.331868132
	MF	GO:0019825~oxygen binding	5	0.015269157	5.013736264
	MF	GO:0008430~selenium binding	3	0.017021027	13.53708791
	MF	GO:0003735~structural constituent of ribosome	15	0.024242368	1.920154314
	MF	GO:0036094~small molecule binding	3	0.027334622	10.82967033
	MF	GO:0008395~steroid hydroxylase activity	4	0.031812352	5.553677092
	MF	GO:0031731~CCR6 chemokine receptor binding	4	0.038864099	5.156985871
Paternal line vs. Maternal line	BP	GO:0006955~immune response	22	1.68E-06	3.429341529
	BP	GO:0055085~transmembrane transport	22	5.96E-05	2.722415863
	BP	GO:0045087~innate immune response	13	2.49E-04	3.595918279
	BP	GO:0042446~hormone biosynthetic process	5	2.74E-04	14.10706402
	BP	GO:0051482~positive regulation of cytosolic calcium ion concentration involved in phospholipase C-activating G-protein coupled signaling pathway	5	0.003182625	7.837257788
	BP	GO:0010594~regulation of endothelial cell migration	3	0.003656145	28.21412804
	BP	GO:0043123~positive regulation of I-kappaB kinase/NF-kappaB signaling	9	0.004128984	3.478454141

BP	GO:0006805~xenobiotic metabolic process	7	0.004272738	4.488611278
BP	GO:0042448~progesterone metabolic process	4	0.004378837	11.28565121
BP	GO:0006278~RNA-dependent DNA biosynthetic process	3	0.007141133	21.16059603
BP	GO:1990830~cellular response to leukemia inhibitory factor	5	0.008010722	6.133506095
BP	GO:0006954~inflammatory response	11	0.008485869	2.652610328
BP	GO:0019886~antigen processing and presentation of exogenous peptide antigen via MHC class II	4	0.011956304	8.061179439
BP	GO:0046677~response to antibiotic	4	0.014559304	7.523767476
BP	GO:0006508~proteolysis	18	0.01837831	1.846742926
BP	GO:0050852~T cell receptor signaling pathway	5	0.030969141	4.149136476
BP	GO:0016477~cell migration	10	0.046371705	2.12136301
CC	GO:0005887~integral component of plasma membrane	30	1.50E-05	2.450853099
CC	GO:0016020~membrane	91	1.04E-04	1.473998576
CC	GO:0016021~integral component of membrane	83	9.54E-04	1.408327414
CC	GO:0005576~extracellular region	33	9.73E-04	1.849363357
CC	GO:0005615~extracellular space	45	0.002273453	1.589157398
CC	GO:0042613~MHC class II protein complex	4	0.008162256	9.225262433
CC	GO:0009897~external side of plasma membrane	13	0.015990831	2.189704145
CC	GO:0005886~plasma membrane	74	0.018191549	1.283955796
CC	GO:0005903~brush border	4	0.027398098	5.996420582
CC	GO:0009986~cell surface	12	0.0397072	1.987763729
CC	GO:0043020~NADPH oxidase complex	3	0.049823091	8.176937157
MF	GO:0005506~iron ion binding	15	2.05E-05	4.002436796
MF	GO:0004508~steroid 17-alpha-monooxygenase activity	4	8.29E-04	18.67803838
MF	GO:0047442~17-alpha-hydroxyprogesterone aldolase activity	4	8.29E-04	18.67803838
MF	GO:0022857~transmembrane transporter activity	11	0.001119853	3.502132196
MF	GO:0038023~signaling receptor activity	9	0.007499368	3.151918977
MF	GO:0008395~steroid hydroxylase activity	4	0.009833991	8.620633098
MF	GO:0016712~oxidoreductase activity, acting on paired donors, with incorporation or reduction of molecular oxygen, reduced flavin or flavoprotein as one donor, and incorporation of one atom of oxygen	5	0.011093212	5.603411514
MF	GO:0016491~oxidoreductase activity	10	0.011136349	2.720102677
MF	GO:0020037~heme binding	10	0.013282589	2.643118639
MF	GO:0003964~RNA-directed DNA polymerase activity	3	0.017262867	14.00852878
MF	GO:0004896~cytokine receptor activity	6	0.017729895	3.90935687
MF	GO:0015267~channel activity	4	0.017791222	7.004264392
MF	GO:0004888~transmembrane signaling receptor activity	7	0.018099278	3.324057678
MF	GO:0005507~copper ion binding	5	0.020909703	4.669509595
MF	GO:0004715~non-membrane spanning protein tyrosine kinase activity	5	0.031678096	4.120155525
MF	GO:0003953~NAD+ nucleosidase activity	4	0.041826884	5.094010467

	MF	GO:0004497~monooxygenase activity	4	0.046869109	4.872531751
Enhancing dominance	BP	GO:0036089~cleavage furrow formation	2	0.023248476	83.53594771
	BP	GO:0045010~actin nucleation	2	0.030879616	62.65196078
	CC	-	-	-	-
	MF	-	-	-	-
Suppressing dominance	BP	GO:0008284~positive regulation of cell proliferation	6	8.83E-04	7.918017553
	BP	GO:0010628~positive regulation of gene expression	5	0.008395575	6.144711538
	BP	GO:0051781~positive regulation of cell division	3	0.008580156	21.06758242
	BP	GO:0060394~negative regulation of pathway-restricted SMAD protein phosphorylation	2	0.014948365	131.0871795
	BP	GO:0007605~sensory perception of sound	3	0.0186868	14.04505495
	BP	GO:0000902~cell morphogenesis	3	0.027875845	11.34408284
	BP	GO:0009887~animal organ morphogenesis	3	0.027875845	11.34408284
	BP	GO:0016477~cell migration	4	0.028961634	5.913707345
	BP	GO:0050768~negative regulation of neurogenesis	2	0.034537827	56.18021978
	BP	GO:0090090~negative regulation of canonical Wnt signaling pathway	3	0.045518689	8.674886878
	CC	-	-	-	-
	MF	GO:0003755~peptidyl-prolyl cis-trans isomerase activity	3	0.014461102	16.08979592
	MF	GO:0004896~cytokine receptor activity	3	0.021364995	13.09634551
Over-dominance	BP	GO:0006413~translational initiation	3	0.023098547	12.57972441
	BP	GO:0006357~regulation of transcription from RNA polymerase II promoter	17	0.026043815	1.785848142
	CC	GO:0034451~centriolar satellite	3	0.037864602	9.653301321
	CC	GO:1990909~Wnt signalosome	2	0.043261199	45.0487395
	CC	GO:0005856~cytoskeleton	6	0.04567523	3.057606753
	MF	GO:0005515~protein binding	32	0.004970608	1.627849356
	MF	GO:0000978~RNA polymerase II core promoter proximal region sequence-specific DNA binding	14	0.020976336	2.007245112
	MF	GO:0000981~RNA polymerase II transcription factor activity, sequence-specific DNA binding	14	0.026595522	1.94214527
	MF	GO:0004040~amidase activity	2	0.028718256	68.4375
	MF	GO:0017064~fatty acid amide hydrolase activity	2	0.047407635	41.0625
	MF	GO:0005198~structural molecule activity	4	0.047741299	4.888392857
Under-dominance	BP	GO:0006412~translation	11	2.22E-06	7.384756802
	BP	GO:0032981~mitochondrial respiratory chain complex I assembly	7	3.00E-06	16.74157934
	BP	GO:0000122~negative regulation of transcription from RNA polymerase II promoter	15	2.24E-05	3.999979136
	BP	GO:0006397~mRNA processing	7	1.92E-04	8.242008291
	BP	GO:0008380~RNA splicing	5	0.003690363	7.809483075

BP	GO:0003009~skeletal muscle contraction	3	0.005685356	25.51097804
BP	GO:0006357~regulation of transcription from RNA polymerase II promoter	23	0.006305615	1.837429525
BP	GO:0006260~DNA replication	5	0.007609041	6.377744511
BP	GO:0006281~DNA repair	6	0.008591153	4.733995926
BP	GO:0009408~response to heat	4	0.012098252	8.273830717
BP	GO:0006457~protein folding	5	0.021076363	4.724255193
BP	GO:0070507~regulation of microtubule cytoskeleton organization	3	0.022384332	12.75548902
BP	GO:0042026~protein refolding	3	0.024807287	12.08414749
BP	GO:0010468~regulation of gene expression	5	0.025606362	4.449589194
BP	GO:0030433~ubiquitin-dependent ERAD pathway	4	0.025633196	6.24758646
BP	GO:0008286~insulin receptor signaling pathway	3	0.029955405	10.9332763
BP	GO:0006406~mRNA export from nucleus	3	0.038388738	9.566616766
BP	GO:0009952~anterior/posterior pattern specification	4	0.042976959	5.102195609
BP	GO:0000398~mRNA splicing, via spliceosome	5	0.045119627	3.715190977
CC	GO:0005634~nucleus	69	4.94E-08	1.858590528
CC	GO:0005654~nucleoplasm	32	1.08E-07	2.916450187
CC	GO:0005747~mitochondrial respiratory chain complex I	8	6.62E-07	15.49364162
CC	GO:0005840~ribosome	9	8.98E-05	6.281206062
CC	GO:0005829~cytosol	33	8.89E-04	1.820833951
CC	GO:0005730~nucleolus	11	0.004151438	2.948616917
CC	GO:0008180~COP9 signalosome	3	0.015408038	15.49364162
CC	GO:0000792~heterochromatin	3	0.017462233	14.52528902
CC	GO:0005739~mitochondrion	14	0.036569393	1.866703809
CC	GO:0005743~mitochondrial inner membrane	6	0.039010515	3.205581025
CC	GO:0016607~nuclear speck	6	0.039010515	3.205581025
CC	GO:0001650~fibrillar center	4	0.048993032	4.841763006
MF	GO:0003723~RNA binding	19	1.91E-05	3.264425529
MF	GO:0003677~DNA binding	20	4.37E-05	2.94622137
MF	GO:0003735~structural constituent of ribosome	10	5.06E-05	5.86109996
MF	GO:0003682~chromatin binding	11	1.23E-04	4.661828737
MF	GO:0051082~unfolded protein binding	7	1.91E-04	8.264150943
MF	GO:0051087~chaperone binding	6	3.89E-04	9.535558781
MF	GO:0051879~Hsp90 protein binding	4	0.001935013	15.74123989
MF	GO:0030544~Hsp70 protein binding	4	0.002868025	13.77358491
MF	GO:0003729~mRNA binding	8	0.004050245	3.958874703
MF	GO:0000978~RNA polymerase II core promoter proximal region sequence-specific DNA binding	18	0.005616122	2.077579846
MF	GO:0003713~transcription coactivator activity	6	0.008355888	4.76777939
MF	GO:0003690~double-stranded DNA binding	4	0.015757985	7.512864494
MF	GO:0015459~potassium channel regulator activity	3	0.025977808	11.80592992

MF	GO:0030911~TPR domain binding	2	0.035643723	55.09433962
MF	GO:0048306~calcium-dependent protein binding	3	0.038668532	9.535558781

' – ' means no significantly enriched was observed in the present gene set.

Table S3. Significantly enriched pathways of different gene sets in the breast muscle.

Gene set	Pathway	Gene count	p-value	Fold enrichment
Maternal line vs. F1 cross	gga04512:ECM-receptor interaction	13	0.006112063	2.43922086
	gga03010:Ribosome	16	0.010593163	2.042424529
	gga03250:Viral life cycle - HIV-1	8	0.042162606	2.442899927
	gga03320:PPAR signaling pathway	9	0.046223775	2.224783862
Paternal line vs. F1 cross	gga02010:ABC transporters	8	0.009244764	3.295121951
	gga03320:PPAR signaling pathway	10	0.010920518	2.680555556
	gga03010:Ribosome	15	0.012164707	2.076331967
Paternal line vs. Maternal line	gga04145:Phagosome	15	4.57E-04	2.96336916
	gga04512:ECM-receptor interaction	10	0.002621141	3.356104832
	gga01100:Metabolic pathways	66	0.005143418	1.342932233
	gga00380:Tryptophan metabolism	6	0.009491695	4.517135692
	gga00260:Glycine, serine and threonine metabolism	6	0.009491695	4.517135692
	gga04148:Efferocytosis	12	0.013451261	2.321305842
	gga00120:Primary bile acid biosynthesis	4	0.014858365	7.428178694
	gga04672:Intestinal immune network for IgA production	5	0.0380856	3.86884307
Enhancing dominance	-	-	-	-
Suppressing dominance	gga04350:TGF-beta signaling pathway	4	0.025495457	6.054901961
	gga03250:Viral life cycle - HIV-1	3	0.038277518	9.349480969
Over-dominance	-	-	-	-
Under-dominance	gga00190:Oxidative phosphorylation	11	1.10E-06	7.668214654
	gga03010:Ribosome	10	4.06E-05	5.828300259
	gga03013:Nucleocytoplasmic transport	8	2.73E-04	6.116581777
	gga03040:Spliceosome	8	9.89E-04	4.946453089
	gga03015:mRNA surveillance pathway	6	0.003649313	5.688421053
	gga04141:Protein processing in endoplasmic reticulum	7	0.01727869	3.318245614
	gga04150:mTOR signaling pathway	6	0.048844595	2.962719298

' – ' means no significantly enriched pathway was detected in this gene set.

Table S4. Significantly enriched GO terms of different gene sets in the liver.

Gene set	Cat-e-gory	Term	Gene count	*p*-value	Fold En-richment
Mater-nal line vs. F1 cross	BP	GO:0006936~muscle contraction	10	4.46E-05	5.317661743
	BP	GO:0000122~negative regulation of transcription from RNA polymerase II promoter	44	7.20E-05	1.875077939
	BP	GO:0006397~mRNA processing	14	0.002007846	2.634287817
	BP	GO:0006805~xenobiotic metabolic process	11	0.002415507	3.057655502
	BP	GO:0045944~positive regulation of transcription from RNA polymerase II promoter	46	0.00263323	1.562801701
	BP	GO:0030239~myofibril assembly	6	0.002657331	5.644902466
	BP	GO:0048536~spleen development	6	0.003858239	5.241695147
	BP	GO:0051897~positive regulation of protein kinase B signal-ing	9	0.004232471	3.335624184
	BP	GO:0007519~skeletal muscle tissue development	7	0.005454323	4.076874003
	BP	GO:0008380~RNA splicing	11	0.005516838	2.745649839
	BP	GO:0006749~glutathione metabolic process	8	0.006123765	3.494463431
	BP	GO:0009952~anterior/posterior pattern specification	12	0.008554739	2.446124402
	BP	GO:0031204~posttranslational protein targeting to mem-brane, translocation	4	0.009005561	8.153748006
	BP	GO:0001709~cell fate determination	4	0.009005561	8.153748006
	BP	GO:0006412~translation	18	0.010968807	1.931150844
	BP	GO:0006281~DNA repair	16	0.011845394	2.017422187
	BP	GO:0002181~cytoplasmic translation	8	0.013083877	3.057655502
	BP	GO:0001756~somitogenesis	7	0.013447089	3.424574163
	BP	GO:0042127~regulation of cell proliferation	12	0.013788216	2.293241627
	BP	GO:0006357~regulation of transcription from RNA polymer-ase II promoter	98	0.014721606	1.251149224
	BP	GO:0031954~positive regulation of protein autophosphory-lation	4	0.014809328	6.988926863
	BP	GO:0043066~negative regulation of apoptotic process	20	0.016825608	1.772553914
	BP	GO:0001837~epithelial to mesenchymal transition	5	0.017400786	4.704085388
	BP	GO:0006635~fatty acid beta-oxidation	7	0.019551423	3.170902002
	BP	GO:0016055~Wnt signaling pathway	12	0.021140813	2.158345061
	BP	GO:0035721~intraciliary retrograde transport	4	0.022272445	6.115311005
	BP	GO:0000381~regulation of alternative mRNA splicing, via spliceosome	9	0.024403264	2.501718138
	BP	GO:0048704~embryonic skeletal system morphogenesis	7	0.027308842	2.952219106
	BP	GO:0061512~protein localization to cilium	5	0.029150575	4.076874003

BP	GO:0010718~positive regulation of epithelial to mesenchymal transition	6	0.02941453	3.335624184
BP	GO:0030198~extracellular matrix organization	13	0.030978306	1.962939335
BP	GO:0043525~positive regulation of neuron apoptotic process	4	0.031412274	5.435832004
BP	GO:0045736~negative regulation of cyclin-dependent protein serine/threonine kinase activity	4	0.031412274	5.435832004
BP	GO:0008284~positive regulation of cell proliferation	20	0.034436012	1.641694229
BP	GO:0060021~palate development	6	0.035136234	3.190597046
BP	GO:0032760~positive regulation of tumor necrosis factor production	6	0.035136234	3.190597046
BP	GO:0003406~retinal pigment epithelium development	3	0.03578244	9.172966507
BP	GO:0035195~gene silencing by miRNA	3	0.03578244	9.172966507
BP	GO:0000398~mRNA splicing, via spliceosome	15	0.040237873	1.781158545
BP	GO:0042733~embryonic digit morphogenesis	6	0.041498437	3.057655502
BP	GO:0001654~eye development	5	0.044641572	3.597241768
BP	GO:0007155~cell adhesion	21	0.048307468	1.556624619
CC	GO:0031012~extracellular matrix	26	1.80E-04	2.255177591
CC	GO:0030016~myofibril	8	0.001020664	4.668059909
CC	GO:0005840~ribosome	19	0.002216868	2.197352525
CC	GO:0005634~nucleus	260	0.003549572	1.160522544
CC	GO:0090575~RNA polymerase II transcription factor complex	10	0.010722525	2.674409323
CC	GO:0000785~chromatin	19	0.014787469	1.833880679
CC	GO:0005844~polysome	6	0.020128575	3.667761357
CC	GO:0005581~collagen trimer	7	0.022081512	3.098625974
CC	GO:0005615~extracellular space	83	0.023511064	1.254987838
CC	GO:0030018~Z disc	10	0.030876597	2.252134167
CC	GO:0000792~heterochromatin	5	0.031174212	4.011613985
CC	GO:0042788~polysomal ribosome	5	0.031174212	4.011613985
CC	GO:0034663~endoplasmic reticulum chaperone complex	3	0.032656166	9.627873563
CC	GO:0030864~cortical actin cytoskeleton	7	0.034576238	2.808129789
CC	GO:0005654~nucleoplasm	81	0.041043533	1.223306288
CC	GO:0005730~nucleolus	32	0.042396451	1.421416166
CC	GO:0005783~endoplasmic reticulum	45	0.043371837	1.32798256
MF	GO:0005509~calcium ion binding	68	5.62E-04	1.514055749
MF	GO:0008013~beta-catenin binding	11	0.001353196	3.285522697
MF	GO:0045159~myosin II binding	5	0.002360191	7.653774464
MF	GO:0070888~E-box binding	8	0.003063048	3.918732526
MF	GO:0008307~structural constituent of muscle	5	0.006200744	6.123019571
MF	GO:0004364~glutathione transferase activity	7	0.006950108	3.896467
MF	GO:0003682~chromatin binding	27	0.008726147	1.69560542
MF	GO:0003735~structural constituent of ribosome	21	0.010462048	1.82387817

	MF	GO:0001222~transcription corepressor binding	6	0.012491919	4.082013048
	MF	GO:0005523~tropomyosin binding	5	0.01281073	5.102516309
	MF	GO:0003714~transcription corepressor activity	16	0.014027002	1.979157841
	MF	GO:0048306~calcium-dependent protein binding	7	0.016214419	3.297010538
	MF	GO:0005516~calmodulin binding	15	0.018191558	1.975167604
	MF	GO:0097100~supercoiled DNA binding	3	0.018867021	12.24603914
	MF	GO:0003723~RNA binding	52	0.031402029	1.323896124
	MF	GO:0004497~monooxygenase activity	6	0.034977696	3.194618907
	MF	GO:0020037~heme binding	15	0.049023541	1.732930067
Paternal line vs. F1 cross	BP	GO:0006635~fatty acid beta-oxidation	10	2.45E-06	7.81139225
	BP	GO:0000122~negative regulation of transcription from RNA polymerase II promoter	33	5.79E-06	2.425069859
	BP	GO:0006633~fatty acid biosynthetic process	8	1.54E-04	6.489464331
	BP	GO:0048536~spleen development	5	0.00340196	7.532413956
	BP	GO:0030308~negative regulation of cell growth	7	0.004071429	4.47379738
	BP	GO:0006805~xenobiotic metabolic process	8	0.004281436	3.834683468
	BP	GO:0055091~phospholipid homeostasis	4	0.004942781	10.54537954
	BP	GO:0030199~collagen fibril organization	5	0.010915998	5.550199757
	BP	GO:0090336~positive regulation of brown fat cell differentiation	3	0.012593353	15.81806931
	BP	GO:0071230~cellular response to amino acid stimulus	5	0.013144356	5.272689769
	BP	GO:0008584~male gonad development	7	0.013474446	3.515126513
	BP	GO:0042632~cholesterol homeostasis	7	0.013474446	3.515126513
	BP	GO:0010951~negative regulation of endopeptidase activity	6	0.016304697	3.954517327
	BP	GO:0046890~regulation of lipid biosynthetic process	3	0.020333957	12.65445545
	BP	GO:0030855~epithelial cell differentiation	5	0.024841818	4.393908141
	BP	GO:0008585~female gonad development	4	0.025995552	6.025931165
	BP	GO:0034097~response to cytokine	4	0.025995552	6.025931165
	BP	GO:0008380~RNA splicing	7	0.027248295	3.012965582
	BP	GO:0000038~very long-chain fatty acid metabolic process	3	0.029553038	10.54537954
	BP	GO:1901223~negative regulation of NIK/NF-kappaB signaling	3	0.029553038	10.54537954
	BP	GO:0006953~acute-phase response	3	0.040093748	9.038896747
	BP	GO:0031954~positive regulation of protein autophosphorylation	3	0.040093748	9.038896747
	BP	GO:0051781~positive regulation of cell division	5	0.041295727	3.766206978
	BP	GO:0006749~glutathione metabolic process	5	0.041295727	3.766206978
	BP	GO:0001649~osteoblast differentiation	6	0.042969638	3.086452548
	BP	GO:0048704~embryonic skeletal system morphogenesis	5	0.046175326	3.636337772
	CC	GO:0005615~extracellular space	72	3.62E-07	1.86322192
	CC	GO:0031012~extracellular matrix	18	3.98E-04	2.672086841
	CC	GO:0005576~extracellular region	39	0.004260237	1.601587253

	CC	GO:0070062~extracellular exosome	9	0.010671263	2.951260093
	CC	GO:0034364~high-density lipoprotein particle	3	0.018811262	13.18229508
	CC	GO:0045277~respiratory chain complex IV	3	0.037185069	9.415925059
	CC	GO:0005777~peroxisome	7	0.037792677	2.796244411
	CC	GO:0030018~Z disc	7	0.0438919	2.698130572
	MF	GO:0008083~growth factor activity	13	0.004475269	2.575305292
	MF	GO:0001222~transcription corepressor binding	5	0.007986555	6.053067993
	MF	GO:0008013~beta-catenin binding	7	0.010337131	3.720422279
	MF	GO:0005507~copper ion binding	6	0.010927521	4.358208955
	MF	GO:0003857~3-hydroxyacyl-CoA dehydrogenase activity	3	0.011821192	16.34328358
	MF	GO:0005504~fatty acid binding	5	0.014013688	5.188343994
	MF	GO:0004364~glutathione transferase activity	5	0.01652123	4.952510176
	MF	GO:0001228~transcriptional activator activity, RNA polymerase II transcription regulatory region sequence-specific binding	16	0.025057778	1.864474419
	MF	GO:0008327~methyl-CpG binding	3	0.037750563	9.33901919
	MF	GO:0015078~hydrogen ion transmembrane transporter activity	4	0.046751616	4.842454395
	MF	GO:0045159~myosin II binding	3	0.048831631	8.171641791
	MF	GO:0004861~cyclin-dependent protein serine/threonine kinase inhibitor activity	3	0.048831631	8.171641791
Paternal line vs. Maternal line	BP	GO:0042773~ATP synthesis coupled electron transport	4	1.82E-04	31.21123321
	BP	GO:0006120~mitochondrial electron transport, NADH to ubiquinone	5	0.001084612	10.64019314
	BP	GO:0061844~antimicrobial humoral immune response mediated by antimicrobial peptide	4	0.006138659	10.4037444
	BP	GO:0006952~defense response	5	0.006253027	6.688121402
	BP	GO:0009952~anterior/posterior pattern specification	6	0.008859288	4.681684982
	BP	GO:0006936~muscle contraction	4	0.012321379	8.142060838
	BP	GO:0051146~striated muscle cell differentiation	3	0.014717896	15.60561661
	BP	GO:0046834~lipid phosphorylation	3	0.014717896	15.60561661
	BP	GO:0035556~intracellular signal transduction	13	0.016017923	2.18927715
	BP	GO:0006281~DNA repair	7	0.017338672	3.378535554
	BP	GO:0042116~macrophage activation	3	0.018141395	14.04505495
	BP	GO:0046339~diacylglycerol metabolic process	3	0.018141395	14.04505495
	BP	GO:0050829~defense response to Gram-negative bacterium	4	0.019122668	6.935829602
	BP	GO:0051216~cartilage development	4	0.019122668	6.935829602
	BP	GO:0022904~respiratory electron transport chain	3	0.021864863	12.76823177
	BP	GO:0019835~cytolysis	3	0.021864863	12.76823177
	BP	GO:0032981~mitochondrial respiratory chain complex I assembly	4	0.03001266	5.852106227
	BP	GO:0007205~protein kinase C-activating G-protein coupled	3	0.030155286	10.80388842

		receptor signaling pathway			
	BP	GO:0051897~positive regulation of protein kinase B signaling	4	0.032508771	5.674769675
	BP	GO:0030900~forebrain development	3	0.034695184	10.0321821
	BP	GO:0006468~protein phosphorylation	16	0.035233922	1.783499041
	BP	GO:0032332~positive regulation of chondrocyte differentiation	3	0.039480909	9.363369963
	BP	GO:0044342~type B pancreatic cell proliferation	2	0.042111903	46.81684982
	BP	GO:0051673~membrane disruption in other organism	2	0.042111903	46.81684982
	BP	GO:0072498~embryonic skeletal joint development	2	0.042111903	46.81684982
	CC	GO:0070469~respiratory chain	6	1.63E-06	25.92263056
	CC	GO:0005615~extracellular space	35	2.08E-04	1.959209416
	CC	GO:0005743~mitochondrial inner membrane	11	9.60E-04	3.605331377
	CC	GO:0005576~extracellular region	23	0.002050693	2.043123219
	CC	GO:0033018~sarcoplasmic reticulum lumen	3	0.002556102	35.64361702
	CC	GO:0005747~mitochondrial respiratory chain complex I	5	0.009555574	5.940602837
	MF	GO:0008137~NADH dehydrogenase (ubiquinone) activity	6	2.13E-05	16.50408206
	MF	GO:0005178~integrin binding	7	0.004576124	4.483985765
	MF	GO:0008083~growth factor activity	8	0.009132432	3.400841152
	MF	GO:0048306~calcium-dependent protein binding	4	0.017326167	7.194087052
	MF	GO:0004143~diacylglycerol kinase activity	3	0.021919311	12.75315432
	MF	GO:0045505~dynein intermediate chain binding	4	0.023241311	6.44987115
	MF	GO:0005507~copper ion binding	4	0.025426579	6.234875445
	MF	GO:0001530~lipopolysaccharide binding	3	0.039576009	9.352313167
	MF	GO:0004674~protein serine/threonine kinase activity	10	0.041020873	2.174956551
	MF	GO:0004672~protein kinase activity	8	0.044174367	2.461135044
	MF	GO:0005509~calcium ion binding	19	0.047084573	1.615399547
	MF	GO:0004553~hydrolase activity, hydrolyzing O-glycosyl compounds	3	0.049858323	8.25204103
Enhancing dominance	BP	GO:0031167~rRNA methylation	4	0.002904465	13.04183673
	BP	GO:0070536~protein K63-linked deubiquitination	4	0.009830315	8.694557823
	BP	GO:0006396~RNA processing	5	0.019385576	4.794792917
	BP	GO:0045197~establishment or maintenance of epithelial cell apical/basal polarity	4	0.025103996	6.210398445
	BP	GO:0017148~negative regulation of translation	5	0.025711573	4.406025924
	BP	GO:0051145~smooth muscle cell differentiation	3	0.029147483	10.86819728
	BP	GO:0018105~peptidyl-serine phosphorylation	8	0.033568879	2.608367347
	BP	GO:0006888~ER to Golgi vesicle-mediated transport	7	0.033776489	2.889014467
	BP	GO:0032727~positive regulation of interferon-alpha production	3	0.035708238	9.781377551
	BP	GO:0001934~positive regulation of protein phosphorylation	6	0.038411008	3.207009033
	BP	GO:0051894~positive regulation of focal adhesion assembly	3	0.042775923	8.89216141

	BP	GO:0035904~aorta development	3	0.042775923	8.89216141
	CC	GO:0005829~cytosol	67	7.47E-05	1.619124382
	CC	GO:0005654~nucleoplasm	40	0.003768035	1.596664185
	CC	GO:0005739~mitochondrion	29	0.007131555	1.693535807
	CC	GO:0030117~membrane coat	4	0.017219926	7.142971352
	CC	GO:0031313~extrinsic component of endosome membrane	3	0.021465237	12.72341772
	CC	GO:1990904~ribonucleoprotein complex	7	0.024178547	3.125049967
	CC	GO:0005622~intracellular	5	0.026912913	4.349886401
	CC	GO:0005737~cytoplasm	95	0.048291803	1.180683452
	MF	GO:0003723~RNA binding	32	2.01E-05	2.324943602
	MF	GO:0005524~ATP binding	50	0.00157391	1.564315511
	MF	GO:0030371~translation repressor activity	4	0.005329163	10.75286416
	MF	GO:0035091~phosphatidylinositol binding	7	0.015659459	3.445459994
	MF	GO:0016853~isomerase activity	3	0.020295885	13.10505319
	MF	GO:0004843~thiol-dependent ubiquitin-specific protease activity	7	0.02125528	3.218784994
	MF	GO:0016491~oxidoreductase activity	8	0.027925229	2.714315224
	MF	GO:0004674~protein serine/threonine kinase activity	12	0.044427243	1.950519545
	MF	GO:0008289~lipid binding	6	0.046887378	3.038852914
	MF	GO:0045505~dynein intermediate chain binding	4	0.048721968	4.82024945
Suppressing dominance	BP	GO:0006412~translation	13	9.38E-06	5.060702973
	BP	GO:0001525~angiogenesis	10	1.55E-04	5.043008207
	BP	GO:0048536~spleen development	4	0.003394183	12.67956349
	BP	GO:0010951~negative regulation of endopeptidase activity	5	0.005456158	6.934136285
	BP	GO:0032729~positive regulation of interferon-gamma production	4	0.012580281	8.068813131
	BP	GO:0002250~adaptive immune response	5	0.013131987	5.412008808
	BP	GO:0008584~male gonad development	5	0.014264204	5.283151455
	BP	GO:0030324~lung development	4	0.015999193	7.396412037
	BP	GO:0032092~positive regulation of protein binding	4	0.019889001	6.827457265
	BP	GO:0032148~activation of protein kinase B activity	3	0.033292808	10.2411859
	BP	GO:0097009~energy homeostasis	3	0.033292808	10.2411859
	BP	GO:0002181~cytoplasmic translation	4	0.0344021	5.547309028
	BP	GO:0001503~ossification	4	0.0344021	5.547309028
	BP	GO:0032981~mitochondrial respiratory chain complex I assembly	4	0.0344021	5.547309028
	BP	GO:0060173~limb development	4	0.0344021	5.547309028
	BP	GO:0008585~female gonad development	3	0.038275913	9.509672619
	BP	GO:0007507~heart development	6	0.038780449	3.208082329
	BP	GO:0030216~keratinocyte differentiation	3	0.043522537	8.875694444
	BP	GO:0032332~positive regulation of chondrocyte differentiation	3	0.043522537	8.875694444

	BP	GO:0070374~positive regulation of ERK1 and ERK2 cascade	6	0.044106255	3.096172481
	BP	GO:0006954~inflammatory response	7	0.048229621	2.65512227
	CC	GO:0005840~ribosome	15	1.16E-07	6.223646327
	CC	GO:0022625~cytosolic large ribosomal subunit	9	4.69E-06	9.210996564
	CC	GO:0005615~extracellular space	40	6.47E-06	2.169846069
	CC	GO:0005576~extracellular region	24	0.001383759	2.066017921
	CC	GO:0005764~lysosome	8	0.010369488	3.319278041
	CC	GO:0005747~mitochondrial respiratory chain complex I	5	0.010641182	5.756872852
	CC	GO:0014704~intercalated disc	3	0.018722894	13.81649485
	CC	GO:0022627~cytosolic small ribosomal subunit	4	0.02883545	5.942578428
	CC	GO:0030018~Z disc	5	0.034644413	4.039910773
	MF	GO:0003735~structural constituent of ribosome	19	7.35E-10	6.462183569
	MF	GO:0004867~serine-type endopeptidase inhibitor activity	7	0.002080626	5.245209854
	MF	GO:0003723~RNA binding	18	0.02323435	1.794618875
	MF	GO:0019834~phospholipase A2 inhibitor activity	2	0.041122407	47.95620438
	MF	GO:0004859~phospholipase inhibitor activity	2	0.041122407	47.95620438
	MF	GO:0031683~G-protein beta/gamma-subunit complex binding	3	0.042609343	8.991788321
Over-dominance	BP	GO:0006635~fatty acid beta-oxidation	13	4.39E-08	7.282621083
	BP	GO:0030488~tRNA methylation	7	0.001048769	5.572531922
	BP	GO:0044208~'de novo' AMP biosynthetic process	4	0.002594158	12.10035503
	BP	GO:0007031~peroxisome organization	4	0.004933961	10.08362919
	BP	GO:1990542~mitochondrial transmembrane transport	4	0.004933961	10.08362919
	BP	GO:0009062~fatty acid catabolic process	4	0.008212616	8.643110735
	BP	GO:1905515~non-motile cilium assembly	6	0.010390025	4.321555368
	BP	GO:0015031~protein transport	16	0.020154758	1.905567721
	BP	GO:0098789~pre-mRNA cleavage required for polyadenylation	3	0.023895814	11.34408284
	BP	GO:0051290~protein heterotetramerization	3	0.023895814	11.34408284
	BP	GO:0006432~phenylalanyl-tRNA aminoacylation	3	0.023895814	11.34408284
	BP	GO:0042760~very long-chain fatty acid catabolic process	3	0.023895814	11.34408284
	BP	GO:0007033~vacuole organization	3	0.023895814	11.34408284
	BP	GO:0006914~autophagy	8	0.026912452	2.688967784
	BP	GO:0006298~mismatch repair	5	0.027455209	4.201512163
	BP	GO:0006606~protein import into nucleus	9	0.029716118	2.430874894
	BP	GO:0016192~vesicle-mediated transport	14	0.033077095	1.890680473
	BP	GO:0009306~protein secretion	6	0.034306184	3.241166526
	BP	GO:0031119~tRNA pseudouridine synthesis	3	0.038096917	9.075266272
	BP	GO:0007127~meiosis I	3	0.038096917	9.075266272
	BP	GO:0006811~ion transport	7	0.041095587	2.714823244
	CC	GO:0005739~mitochondrion	102	7.46E-21	2.723202333
	CC	GO:0005743~mitochondrial inner membrane	35	2.66E-11	3.744173052

	CC	GO:0042645~mitochondrial nucleoid	8	7.61E-05	6.894032922
	CC	GO:0005777~peroxisome	12	6.48E-04	3.384343434
	CC	GO:0005741~mitochondrial outer membrane	13	7.98E-04	3.102314815
	CC	GO:0005759~mitochondrial matrix	16	0.001776339	2.481851852
	CC	GO:0005730~nucleolus	33	0.001876934	1.771217801
	CC	GO:0005778~peroxisomal membrane	7	0.0052985	4.17619302
	CC	GO:0016592~mediator complex	8	0.00842963	3.353853854
	CC	GO:0070209~ASTRA complex	3	0.011893753	15.51157407
	CC	GO:0005829~cytosol	110	0.020687985	1.215294265
	CC	GO:0005751~mitochondrial respiratory chain complex IV	5	0.02070288	4.562227669
	CC	GO:0000176~nuclear exosome (RNase complex)	4	0.022691616	6.20462963
	CC	GO:1990130~Iml1 complex	3	0.022774194	11.63368056
	CC	GO:0045252~oxoglutarate dehydrogenase complex	3	0.022774194	11.63368056
	CC	GO:0005789~endoplasmic reticulum membrane	32	0.024194885	1.490601713
	CC	GO:0034098~VCP-NPL4-UFD1 AAA ATPase complex	3	0.036349164	9.306944444
	CC	GO:0005847~mRNA cleavage and polyadenylation specificity factor complex	4	0.037790808	5.170524691
	MF	GO:0050660~flavin adenine dinucleotide binding	17	1.78E-08	5.598075333
	MF	GO:0010181~FMN binding	7	8.22E-05	8.333786355
	MF	GO:0005524~ATP binding	100	6.40E-04	1.385589239
	MF	GO:0016887~ATPase activity	33	0.001164913	1.824078748
	MF	GO:0005515~protein binding	160	0.003213177	1.227118478
	MF	GO:0017056~structural constituent of nuclear pore	6	0.006013595	4.887483727
	MF	GO:0008757~S-adenosylmethionine-dependent methyltransferase activity	9	0.007437892	3.09540636
	MF	GO:0015075~ion transmembrane transporter activity	4	0.00770157	8.844018173
	MF	GO:0000049~tRNA binding	9	0.009688546	2.963686941
	MF	GO:0070403~NAD+ binding	5	0.010232558	5.527511358
	MF	GO:0003988~acetyl-CoA C-acyltransferase activity	3	0.011945024	15.4770318
	MF	GO:0003676~nucleic acid binding	22	0.014509491	1.746126665
	MF	GO:0016491~oxidoreductase activity	14	0.015025996	2.103674226
	MF	GO:0005324~long-chain fatty acid transporter activity	4	0.016769288	6.878680801
	MF	GO:0008137~NADH dehydrogenase (ubiquinone) activity	5	0.020853744	4.552068177
	MF	GO:0030170~pyridoxal phosphate binding	8	0.02150421	2.814005782
	MF	GO:0004826~phenylalanine-tRNA ligase activity	3	0.022870168	11.60777385
	MF	GO:0000175~3'-5'-exoribonuclease activity	5	0.030666017	4.072903106
	MF	GO:0005506~iron ion binding	13	0.037800842	1.916203937
	MF	GO:0051539~4 iron, 4 sulfur cluster binding	6	0.046489371	2.995554542
Underdominance	BP	GO:0000122~negative regulation of transcription from RNA polymerase II promoter	29	5.66E-08	3.302966574
	BP	GO:0008380~RNA splicing	9	1.05E-04	6.003914609
	BP	GO:0006397~mRNA processing	10	1.47E-04	5.028919929

BP	GO:0006412~translation	13	1.84E-04	3.727576614
BP	GO:0030168~platelet activation	5	2.38E-04	14.85817252
BP	GO:0045944~positive regulation of transcription from RNA polymerase II promoter	25	2.86E-04	2.269998579
BP	GO:0030433~ubiquitin-dependent ERAD pathway	8	6.63E-04	5.336812986
BP	GO:0048536~spleen development	5	6.70E-04	11.67427841
BP	GO:0051258~protein polymerization	4	9.01E-04	18.67884545
BP	GO:0006281~DNA repair	10	0.00280323	3.369894798
BP	GO:0071230~cellular response to amino acid stimulus	5	0.002805927	8.171994885
BP	GO:0006457~protein folding	9	0.00322461	3.631997727
BP	GO:0010718~positive regulation of epithelial to mesenchymal transition	5	0.004037439	7.429086259
BP	GO:0006880~intracellular sequestering of iron ion	3	0.005349713	24.51598465
BP	GO:0006325~chromatin organization	8	0.008977821	3.396153718
BP	GO:0006749~glutathione metabolic process	5	0.009788596	5.837139204
BP	GO:0006364~rRNA processing	6	0.011564626	4.358397272
BP	GO:0048103~somatic stem cell division	3	0.012841689	16.34398977
BP	GO:0001654~eye development	4	0.013946253	7.691289303
BP	GO:0045892~negative regulation of transcription, DNA-templated	11	0.015297827	2.429511993
BP	GO:0002181~cytoplasmic translation	5	0.015629892	5.107496803
BP	GO:0007596~blood coagulation	5	0.015629892	5.107496803
BP	GO:0051897~positive regulation of protein kinase B signaling	5	0.017368489	4.952724173
BP	GO:0090314~positive regulation of protein targeting to membrane	3	0.023021434	12.25799233
BP	GO:0016573~histone acetylation	4	0.024937585	6.226281817
BP	GO:0043066~negative regulation of apoptotic process	10	0.025511648	2.36869417
BP	GO:0045893~positive regulation of transcription, DNA-templated	12	0.026545759	2.120301376
BP	GO:0030970~retrograde protein transport, ER to cytosol	3	0.035540444	9.806393862
BP	GO:0006826~iron ion transport	3	0.035540444	9.806393862
BP	GO:0001649~osteoblast differentiation	5	0.035585505	3.986338968
BP	GO:0006334~nucleosome assembly	5	0.035585505	3.986338968
BP	GO:0006260~DNA replication	6	0.035796337	3.268797954
BP	GO:0000398~mRNA splicing, via spliceosome	8	0.038050212	2.538872197
BP	GO:0060325~face morphogenesis	3	0.042577088	8.914903511
BP	GO:0006465~signal peptide processing	3	0.042577088	8.914903511
BP	GO:0006338~chromatin remodeling	6	0.042830655	3.113140909
BP	GO:0009615~response to virus	4	0.043617861	5.028919929
BP	GO:0042127~regulation of cell proliferation	6	0.045347317	3.064498082
BP	GO:0008285~negative regulation of cell proliferation	8	0.047179639	2.421331818

CC	GO:0005788~endoplasmic reticulum lumen	10	5.27E-07	9.684925567
CC	GO:0005634~nucleus	121	6.21E-05	1.385388866
CC	GO:0005654~nucleoplasm	47	8.90E-05	1.820766007
CC	GO:0005730~nucleolus	22	1.96E-04	2.506686617
CC	GO:0005840~ribosome	12	5.38E-04	3.559864533
CC	GO:0000792~heterochromatin	5	0.001130644	10.29023342
CC	GO:0005615~extracellular space	42	0.002054596	1.628983947
CC	GO:0000785~chromatin	12	0.002376954	2.97101476
CC	GO:0005577~fibrinogen complex	3	0.002691395	32.92874693
CC	GO:0005783~endoplasmic reticulum	25	0.003485818	1.89245672
CC	GO:0005787~signal peptidase complex	3	0.008615635	19.75724816
CC	GO:0071011~precatalytic spliceosome	5	0.009552732	-
MF	GO:0003682~chromatin binding	21	8.31E-07	3.743589744
MF	GO:0003723~RNA binding	31	5.57E-05	2.24037224
MF	GO:0042393~histone binding	11	2.27E-04	4.296415195
MF	GO:0003677~DNA binding	32	3.73E-04	1.982853748
MF	GO:0003735~structural constituent of ribosome	13	7.48E-04	3.204998311
MF	GO:0031386~protein tag	4	0.002419471	13.9047619
MF	GO:0001228~transcriptional activator activity, RNA polymerase II transcription regulatory region sequence-specific binding	14	0.002898389	2.602495544
MF	GO:0003755~peptidyl-prolyl cis-trans isomerase activity	6	0.003024942	5.959183673
MF	GO:0004364~glutathione transferase activity	5	0.003237998	7.9004329
MF	GO:0001221~transcription cofactor binding	4	0.006739148	9.931972789
MF	GO:0001046~core promoter sequence-specific DNA binding	4	0.011813583	8.179271709
MF	GO:0051082~unfolded protein binding	7	0.015032229	3.476190476
MF	GO:0042826~histone deacetylase binding	6	0.02504802	3.596059113
MF	GO:0030544~Hsp70 protein binding	4	0.030346487	5.793650794
MF	GO:0031492~nucleosomal DNA binding	4	0.033771034	5.561904762
MF	GO:0002020~protease binding	4	0.033771034	5.561904762
MF	GO:0042802~identical protein binding	17	0.036495408	1.738095238
MF	GO:0008320~protein transmembrane transporter activity	3	0.038050963	9.480519481
MF	GO:0003756~protein disulfide isomerase activity	3	0.038050963	9.480519481

' – ' means no significantly enriched was observed in the present gene set.

Table S5. Significantly enriched pathways of different gene sets in the liver.

**Gene set**	**Pathway**	**Gene count**	**p-value**	**Fold enrichment**
Maternal line vs. F1 cross	gga03060:Protein export	8	0.003323397	3.832624113
	gga03010:Ribosome	21	0.003824806	1.97914196
	gga00982:Drug metabolism - cytochrome P450	9	0.006016501	3.135783366

	gga04350:TGF-beta signaling pathway	17	0.018012258	1.861560284
	gga04141:Protein processing in endoplasmic reticulum	22	0.018522714	1.68635461
	gga04310:Wnt signaling pathway	22	0.02422121	1.642553191
	gga00980:Metabolism of xenobiotics by cytochrome P450	8	0.024271945	2.705381727
	gga03320:PPAR signaling pathway	11	0.043150788	2.007565012
Paternal line vs. F1 cross	gga00620:Pyruvate metabolism	12	9.47E-07	6.539733764
	gga00071:Fatty acid degradation	10	2.08E-05	6.110357304
	gga01212:Fatty acid metabolism	12	4.16E-05	4.565474514
	gga00640:Propanoate metabolism	9	7.23E-05	6.049253731
	gga03320:PPAR signaling pathway	12	2.16E-04	3.84079602
	gga00010:Glycolysis / Gluconeogenesis	10	7.49E-04	3.953760609
	gga00650:Butanoate metabolism	6	0.004579296	5.260220636
	gga00280:Valine, leucine and isoleucine degradation	8	0.005283762	3.666214383
	gga01100:Metabolic pathways	87	0.005780398	1.281434319
	gga04910:Insulin signaling pathway	14	0.005944967	2.352487562
	gga04540:Gap junction	11	0.007952329	2.640547264
	gga00061:Fatty acid biosynthesis	5	0.008322471	5.930640913
	gga00380:Tryptophan metabolism	7	0.008772056	3.814844695
	gga04145:Phagosome	15	0.009394343	2.145125437
	gga04216:Ferroptosis	6	0.021614371	3.666214383
	gga00982:Drug metabolism - cytochrome P450	6	0.021614371	3.666214383
	gga00480:Glutathione metabolism	7	0.032293216	2.880597015
Paternal line vs. Maternal line	gga00564:Glycerophospholipid metabolism	8	0.003701393	3.957162471
	gga04010:MAPK signaling pathway	13	0.007777406	2.367778901
	gga04310:Wnt signaling pathway	9	0.01556558	2.746245059
Enhancing dominance	gga00970:Aminoacyl-tRNA biosynthesis	7	0.004299584	4.443035001
	gga00640:Propanoate metabolism	5	0.022345795	4.548821549
	gga00190:Oxidative phosphorylation	9	0.031844352	2.408199643
Suppressing dominance	gga03010:Ribosome	15	5.24E-06	4.429508197
Over-dominance	gga01100:Metabolic pathways	168	8.85E-12	1.575212241
	gga04146:Peroxisome	22	3.32E-07	3.574611383
	gga00640:Propanoate metabolism	12	7.73E-06	5.134441805
	gga01210:2-Oxocarboxylic acid metabolism	11	1.23E-05	5.430659602
	gga01200:Carbon metabolism	22	1.41E-05	2.881574483
	gga00280:Valine, leucine and isoleucine degradation	14	1.75E-05	4.084215072
	gga01240:Biosynthesis of cofactors	22	6.15E-04	2.241224598
	gga00071:Fatty acid degradation	10	6.79E-04	3.88972864
	gga00785:Lipoic acid metabolism	7	0.001255628	5.285454799

	gga00190:Oxidative phosphorylation	18	0.00199254	2.265194914
	gga01212:Fatty acid metabolism	12	0.002103333	2.906287814
	gga00020:Citrate cycle (TCA cycle)	8	0.003684507	3.803290226
	gga00410:beta-Alanine metabolism	8	0.003684507	3.803290226
	gga00970:Aminoacyl-tRNA biosynthesis	10	0.004952504	2.985140584
	gga00260:Glycine, serine and threonine metabolism	9	0.006454666	3.122295692
	gga00270:Cysteine and methionine metabolism	10	0.006777459	2.85246767
	gga03018:RNA degradation	12	0.01638394	2.232366002
	gga00380:Tryptophan metabolism	8	0.02180442	2.775373949
	gga03320:PPAR signaling pathway	11	0.022209763	2.241224598
	gga00650:Butanoate metabolism	6	0.02893685	3.348549003
Under-dominance	gga04141:Protein processing in endoplasmic reticulum	25	2.80E-10	4.690972222
	gga03010:Ribosome	14	3.32E-04	3.229849727
	gga00982:Drug metabolism - cytochrome P450	7	8.99E-04	5.970328283
	gga04350:TGF-beta signaling pathway	11	0.00374327	2.948611111
	gga00980:Metabolism of xenobiotics by cytochrome P450	6	0.006322693	4.966911765
	gga03060:Protein export	5	0.00922394	5.863715278
	gga00480:Glutathione metabolism	6	0.028347564	3.446428571
	gga04310:Wnt signaling pathway	11	0.045215664	2.010416667
	gga03013:Nucleocytoplasmic transport	8	0.045434631	2.421146953
	gga00983:Drug metabolism - other enzymes	6	0.049766749	2.962719298
	gga03040:Spliceosome	9	0.049870275	2.202717391

'–' means no significantly enriched pathway was detected in this gene set.

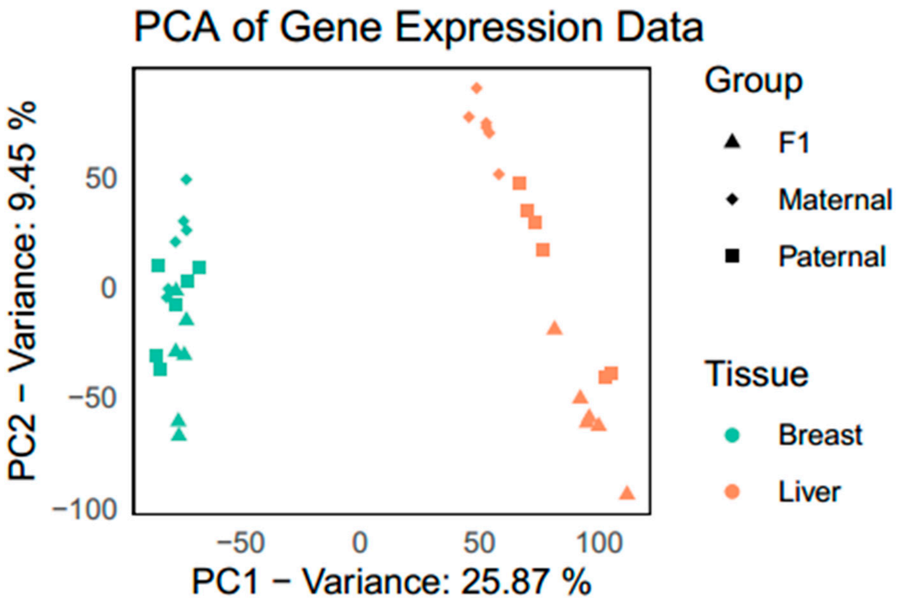

Figure S1. The principal component analysis (PCA) of sequencing data.
